# Effect of Shoot Cutting on Trace Metal Concentration in Leaves and Capitula of Potential Phytoaccumulator, Invasive *Erigeron annuus* (Asteraceae)

**DOI:** 10.1007/s00128-020-02844-7

**Published:** 2020-04-17

**Authors:** Artur Pliszko, Beata Klimek, Kinga Kostrakiewicz-Gierałt

**Affiliations:** 1 Faculty of Biology, Institute of Botany, Jagiellonian University, Gronostajowa 3, 30-387 Kraków, Poland; 2 Faculty of Biology, Institute of Environmental Sciences, Jagiellonian University, Gronostajowa 7, 30-387 Kraków, Poland; 3 Faculty of Tourism and Recreation, University of Physical Education in Krakow, Jana Pawła II 78, 31-571 Kraków, Poland

**Keywords:** Alien species, Phytoremediation, Plant regeneration, Soil pollution, Urban areas

## Abstract

The effect of shoot cutting was tested on cadmium, lead and zinc concentration in leaves and capitula of *Erigeron annuus*, an invasive species, which is considered as a potential phytoremediator. Plant material and soil were collected in the city center of Kraków, southern Poland, considered as one of the most contaminated cities in Europe. We proved that the concentration of zinc in leaves and capitula was higher after regrowth, concentration of cadmium was lower in capitula than in leaves, and the average value of bio-concentration factor for zinc and cadmium was less than 1, whereas for lead it was greater than 1 in both plant organs. Our results suggested that *E*. *annuus* can be potentially used for phytoremediation of lead and cutting the shoots can promote effectiveness of zinc removal from the contaminated soil.

Negative effects of plant invasion such as reduction of native biodiversity, hybridization with native congeners and high costs of controlling of invader populations have been well documented wóorldwide (Powell et al. 2011; Vilà et al. 2011). However, there are numerous findings that some invasive alien plants can facilitate native species (Rodriguez 2006). Alien plants can be useful in ecosystem restoration, especially when native species are locally absent or extinct, or because the alien plants are more effective (Ewel and Putz 2004; Trueman and Erber 2013; Pandey et al. 2015a, b). Revegetation of degraded areas is crucial for maintaining ecological services and sustaining ecosystem functions (Pandey 2012).

Invasive plants can also be used in phytoremediation of heavy metals in polluted and environmentally devastated areas (Dissanayake et al. 2002; Pandey 2012, 2016; Fu et al. 2017). *Erigeron annuus* (L.) Desf. (Asteraceae), an annual, biennial or short-lived perennial plant, is native to eastern North America (Stratton 1991). It has been widely naturalized in Central America, Europe, Asia and Oceania (Randall 2017; Pliszko et al. 2017) and classified as invasive in many European countries (Randall 2017; Sennikov and Kurtto 2019). It is usually found in anthropogenic habitats such as abandoned fields, roadsides, railway embankments and waste ruderal ground (Stratton 1991), showing a high tolerance to disturbance and soil pollution (Liu et al. 2008; Li et al. 2011). According to Mucina (1997), it is a diagnostic species of ruderal communities of temperate and Mediterranean regions of the class *Artemisietea vulgaris*.

*Erigeron annuus* can be potentially used in phytoremediation (Yang et al. 2014; Fu et al. 2016). Phytoremediators are demanded to be characterized by plenteous biomass (Pandey et al. 2015a, b), which is not a case of *E. annuus.* However, after shoot cutting the organs can regenerate within few weeks, which can increase whole-year biomass production and therefore amount of heavy metal retrieved from the soil. Interestingly, Qi et al. (2019) observed that moderate mowing height (stubble height of 15 cm) greatly influenced the plant growth and achieved a significant biomass harvest, thereby resulting in higher uranium uptake in *Lolium multiflorum* Lam. and *L*. *perenne* L. This phenomenon can be essential for metal-contaminated urban areas, where lawn and grasses are mowed few times per year.

In our study we aimed to check effect of shoot cutting on metal concentration in *E. annuus* and therefore, effectiveness of that species for phytoremediation. We expected that metal concentration in regenerated organs will be at least on similar level as before shoot cutting or even higher. We were also intrigued how metals are distributed between plant vegetative and generative organs. Mohsenzadeh et al. (2011) showed that the excessive amounts of heavy metals negatively affect plant growth and development, including the development of the ovules and embryos. Musielińska et al. (2016) showed the lower concentration of zinc in flowers than in leaves of the closely related *E. ramosus* (Walters) Britton, Sterns & Poggenb., as well as in many other species from the family of Asteraceae. Also Wei et al. (2009) showed lower concentration of cadmium in the inflorescences than in the leaves in *E. canadensis* L. Given this, we tested the hypothesis that the concentration of metals in the capitula of *E*. *annuus* is lower than that in its leaves, regardless of the regrowth of the shoots after the cutting.

## Material and Methods

The study was conducted in 2016, in Kraków, the second largest city in Poland, where the average annual air temperature is 9.3°C and the average annual precipitation is 730 mm in the city center (Matuszko and Piotrowicz 2015). Kraków is considered to be one of the most contaminated cities in Europe (Błońska et al. 2016). The study was based on data from one population of *Erigeron annuus* s. str. dispersed on an area of about 500 m^2^. The population occurred on a waste ruderal ground, among railway embankments and busy streets, in the city center (GPS coordinates: 50° 04,053′ N and 19° 58,561′ E; altitude 202 m a.s.l.). Taxonomic treatment of the species followed Sennikov and Kurtto (2019). On June 19, 10 flowering individuals of *E*. *annuus* were randomly selected and marked with plastic labels from 1 to 10. Next, all the shoots of each individual were cut off at a height of 5 cm from the ground level and collected for further investigation. After the regrowth (58 days later), at the stage of flowering, the shoots of each individual were collected once again In the laboratory, the collected shoots of *E. annuus* were washed using distilled water to remove the dust from their surfaces. Next, living and undamaged leaves and capitula were separated from each plant separately and prefatory air-dried at room temperature.

During second plant sampling, the soil samples were collected from the plant’s rhizosphere, under each individual separately (about 300 g). The soil dry weight (DW) was determined after drying the soil samples at 105°C for 24 h. The soil pH was measured potentiometrically in water and in 1 M KCl solution (1:10 w/v). The C and N contents were analysed using a CHNS analyser (Vario EL III, Elementar Analysensysteme GmbH). The total element (Ca, K, Mg, Na, and P) in each soil sample were determined after wet digestion of 0.5 g of DW in 10 ml of SupraPure-concentrated HNO_3_ and HClO_4_ (7:1 v/v) (Sigma-Aldrich). Also plant organs, leaves and capitula were subjected to wet digestion and heavy metals (Zn, Cd and Pb) concentrations were measured both in soil and plant organs. The concentrations of the metals in the digests were measured using atomic absorption spectrometry (AAS) with a flame or graphite furnace nebuliser (Perkin-Elmer), and the P concentration was measured on a flow-injection analyser (FIA compact, MLE). The accuracy of the mineralisation process was assessed using blank samples and samples of standard certified materials (CRM048-050, Sandy Loam 8, RT Corp. and SRM 157a Pine Needles).

Bio-concentration factor (BF) for plant organs for Zn, Cd or Pb was calculated as follows: BF = *Cp*/*Cs*, where *Cp* is the metal concentration in plant organ (capitula or leaves), and *Cs* is the heavy metal concentration in the corresponding rhizosphere soil (Yang et al. 2014). Plants reaching the value of the bio-concentration factor less than 1 are considered to be unsuitable for use in phytoremediation application (Fitz and Wenzel 2002). BF was calculated for plant organs collected after shoot regrowth.

Normal distribution in data was tested using the Shapiro–Wilk test, while homogeneity of variance was tested using the Levene test at the significance level of *p* < 0.05. As the values in majority of groups were not consistent with normal distribution and the variance was not homogenous, the analysis was based on the non-parametric tests. The Wilcoxon matched-pairs test was used to check for the differences Zn, Cd and Pb concentrations as well as for BF in the capitula and leaves of individual plants, separately for plant organs collected before cutting and after shoot regrowth. Analyses were conducted using PAST 2.17c software (Natural History Museum, University of Oslo, Norway).

## Results and Discussion

The soil samples showed an alkaline pH and low content of the biogenic elements, except for Ca (Table 1). A relatively high content of Ca and Mg might resulted from the bedrock rich in alkaline minerals, as Kraków city is located on the Polish Jurassic Highland, geological structure composed of jurassic limestones and dolomites. However, industrial areas substrate may contain elements deposited from different sources, e.g. construction materials. Soils collected in our study were characterized by a high concentration of zinc (on average 1448 mg kg^−1^ DW). This value exceed several times soil Zn concentration accepted for urbanized areas in Poland, that is 300 mg Zn kg^−1^ soil DW and slightly a value accepted for transportation zones, that is 1000 mg Zn kg^−1^ soil DW (Regulation of the Ministry of Environment, Dz.U. 2002 nr 165 poz. 1359). The soil Zn concentration were much higher than values measured by Ciarkowska et al (2019) in urban area of Kraków, which do not exceed 200 mg Zn kg^−1^ DW. Moreover, that author give several examples of cities with comparable soil Zn content (see Ciarkowska et al. 2019). Therefore, high concentration of soil Zn observed in our study suggest that traffic was a local source of contamination.

**Table 1:** Mean values, standard deviations and minimal and maximal values for physical and chemical properties of studied soils (n = 10)

Soil property	Unit	Data set values			
		Mean	SD	Min	Max
pH in water		8.37	0.09	8.21	8.48
pH in KCl		8.17	0.13	7.94	8.34
C	% DW	3.77	2.11	1.79	7.87
N	% DW	0.18	0.08	0.09	0.32
P	% DW	0.56	0.10	0.36	0.67
S	% DW	0.05	0.03	0.01	0.09
Ca	% DW	2.96	0.99	1.24	4.52
K	% DW	0.30	0.08	0.22	0.47
Mg	% DW	0.60	0.26	0.23	1.20
Na	% DW	0.03	0.01	0.02	0.04
Zn	mg kg^−1^ DW	1448	842	226	3161
Cd	mg kg^−1^ DW	2.24	1.16	0.64	3.87
Pb	mg kg^−1^ DW	0.12	0.06	0.01	0.25

Soils of areas strongly transformed by human activity are critical environment for many plant species (Jaźwa et al. 2016). Heavy metals are long-term pollutants, as once incorporated into the soil they remain for very long periods, up to thousands of years (Kabata-Pendias and Pendias 2001). Metal toxicity for organisms depends not only on its total concentration in soil, but also on its biological availability. High soil pH prevent for metal mobility because of reduced solubility of metal salts (Caporale and Violante 2016). In our study we found high soil pH, therefore metal availability for plants was possibly low.

Toxic substance (metal) distribution in plant organs may reflect the hazard for reproduction. Metal content was expected to be lower in capitula than in leaves. In a current study, we showed no difference in zinc concentration between capitula and leaves of *E. annuus*, but cadmium concentration was lower in capitula than in leaves (Table 2). In case of lead, its concentration was lower in capitula than in leaves only after shoot cutting and regrowth (Table 2). Therefore, our hypothesis cannot be fully accepted. Zinc belongs to essential elements, which means that some amounts of this element are necessary for a proper functioning of organisms. Simultaneously, zinc toxicity is moderate, and its increased concentrations are relatively well tolerated by living organisms. In a current study we did not measure metal concentration in seeds, which may provide additional evidence for zinc tolerance by *E. annuus*. Cadmium is known to be more toxic for organisms than zinc (Welp 1999). The lower cadmium concentration in the inflorescences than in the leaves was found also in *E. rigeron canadensis* (Wei et al. 2009).

**Table 2:** The mean content and standard deviations (in parenthesis) for the Zn, Cd and Pb content (mg kg^−1^ soil DW) in the capitula and leaves of *Erigeron annuus*, collected before and after the regrowth of the shoots

	Plant organs (these same plant individual)								
	Capitula			Leaves			Difference between capitula and leaves (*p* value)		
	Zn	Cd	Pb	Zn	Cd	Pb	Zn	Cd	Pb
	mg kg^−1^ soil DW								
Shoot cutting									
Before	78 (7)	0.06 (0.04)	0.20 (0.11)	78 (18)	0.16 (0.08)	0.34 (0.17)	0.878	**0.005**	0.093
After	120 (30)	0.09 (0.04)	0.21 (0.12)	166 (102)	0.26 (0.13)	0.56 (0.31)	0.114	**0.005**	**0.022**
Difference between cutting (p value)	**0.011**	**0.042**	0.374	**0.007**	0.093	**0.017**			

Results of Wilcoxon-matched test were presented; p values for comparisons between selected data sets. Significant differences between groups (*p* < 0.05) were given with bolded font

Metal concentration in plants was higher after regrowth of the shoots comparing to before cutting or was observed to be on a similar level (Table 2). Only zinc concentration was higher for both capitula and leaves after shoot regrowth comparing initial Zn concentration (Table 2). Higher metal concentration in regrowth plants can be explained by the fact that cutting promotes plant compensatory growth and thereby it strengthens the ability of metal removal (Qi et al. 2019). In our experiment, the plants were cut off at the height of 5 cm from the ground level. Presumably, using other cutting height (e.g. stubble height of 15 cm) we could get a higher concentration of heavy metals in leaves and capitula of *E*. *annuus* after regrowth as it was revealed by Qi et al. (2019) in *Lolium multiflorum* and *L*. *perenne*.

Metal concentration in plants is showed to be related to metal concentration in soil/substrate. For example, Xiong et al. (2013) found positive correlation between the concentration of cadmium in substrate and in the aboveground plant organs in *E. breviscapus* (Vaniot) Hanz–Mazz. Bio-concentration factor (BF) reflects not only metal concentration in soil, but also plant tendency to accumulate of metal. In our study, the average value of BF for zinc and cadmium was less than 1, whereas for lead it was greater than 1 in both plant organs (Table 3). This might suggest that *E. annuus* is a phytoremediatior for lead, but not for zinc or cadmium.

**Table 3:** The mean and standard deviations (in parenthesis) for the BF for Zn, Cd and Pb content in the capitula and leaves of *Erigeron annuus*, collected before and after the regrowth of the shoots

Plant organs (these same plant individual)								
Capitula			Leaves			Difference between capitula and leaves (*p* value)		
BF Zn	BF Cd	BF Pb	BF Zn	BF Cd	BF Pb	BF Zn	BF Cd	BF Pb
0.13 (0.11)	0.06 (0.07)	3.01 (3.89)	0.21 (0.24)	0.16 (0.12)	8.57 (10.34)	0.173	**0.005**	**0.017**

Results of Wilcoxon-matched test were presented; *p* values for comparisons between selected data sets. Significant differences between groups (*p* < 0.05) were given with bolded font

Interestingly, Yang et al. (2014) and Fu et al. (2016) considered *E*. *annuus* as a potential phytoremediator for metal and metalloid contaminated soils due to its ability of lead and antimony accumulation. Menzies et al. (2007) argued that lead is a hardly mobile element and it accumulates mainly in the plant roots, whereas the mobility of zinc and especially cadmium is higher and both metals are easily transferred from the roots to the aboveground parts of the plants. Moreover, it is worth mentioning that *E. canadensis* presented the substantial value of BF factor for cadmium (Murariu et al. 2007) and zinc (Vukojević et al. 2016) comparing to other metals. Considering the results presented in this study, we can draw a conclusion that further investigations are needed to establish the potential of *E*. *annuus* for phytoremediation.
